# TMT-Based Quantitative Proteomic Analysis of Intestinal Organoids Infected by *Listeria monocytogenes* Strains with Different Virulence

**DOI:** 10.3390/ijms23116231

**Published:** 2022-06-02

**Authors:** Cong Zhou, Yafang Zou, Jie Huang, Ziyu Zhao, Yanning Zhang, Yeyu Wei, Keping Ye

**Affiliations:** Jiangsu Collaborative Innovation Center of Meat Production and Processing, Quality and Safety Control, College of Food Science and Technology, Nanjing Agricultural University, Nanjing 210095, China; 2019108059@njau.edu.cn (C.Z.); 2021108056@stu.njau.edu.cn (Y.Z.); 2017108082@njau.edu.cn (J.H.); zbzhaoziyu@163.com (Z.Z.); ynzhangnjau@163.com (Y.Z.); yywei0011@163.com (Y.W.)

**Keywords:** *L. monocytogenes*, virulence, proteomic, host response, intestinal organoid

## Abstract

*L. monocytogenes*, consisting of 13 serotypes, is an opportunistic food-borne pathogen that causes different host reactions depending on its serotypes. In this study, highly toxic *L. monocytogenes* *10403s* resulted in more severe infections and lower survival rates. Additionally, to investigate the remodeling of the host proteome by strains exhibiting differential toxicity, the cellular protein responses of intestinal organoids were analyzed using tandem mass tag (TMT) labeling and high-performance liquid chromatography–mass spectrometry. The virulent strain *10403s* caused 102 up-regulated and 52 down-regulated proteins, while the low virulent strain *M7* caused 188 up-regulated and 25 down-regulated proteins. Based on the analysis of gene ontology (GO) and KEGG databases, the expressions of differential proteins in organoids infected by *L. monocytogenes* *10403s* (virulent strain) or *M7* (low virulent strain) were involved in regulating essential processes such as the biological metabolism, the energy metabolism, and immune system processes. The results showed that the immune system process, as the primary host defense response to *L. monocytogenes*, comprised five pathways, including ECM–receptor interaction, the complement and coagulation cascades, HIF-1, ferroptosis, and NOD-like receptor signaling pathways. As for the *L. monocytogenes* *10403s* vs. *M7* group, the expression of differential proteins was involved in two pathways: systemic lupus erythematosus and transcriptional mis-regulation in cancer. All in all, these results revealed that *L. monocytogenes* strains with different toxicity induced similar biological functions and immune responses while having different regulations on differential proteins in the pathway.

## 1. Introduction

*L. monocytogenes* is a Gram-positive food-borne pathogen that can lead to listeriosis, which has a high mortality rate ranging from 20 to 30% [1]. *L. monocytogenes* has 4 evolutionary lineages (I, II, III, and IV) based on multigene phylogenetic analyses, and is divided into 13 serotypes according to somatic O antigen [2]. Of the 13 serotypes, over 98% of isolates from human listeriosis belong to serotypes within lineages I and II (1/2a, 1/2c, 1/2b, and 4b) [3]. In addition, strains belonging to different serovars present with different levels of virulence in the host and cause different host reactions; this is due to differences in growth and in the expression of virulence factors [4,5]. There are many studies on the comparison of *L. monocytogenes* strains with different toxicity, mainly focusing on the following aspects: the genetic relationship between virulent and low-virulence strains [6], survival ability under stressful environments [7,8], biological characteristics and pathogenicity in cell and mouse models [9,10], the expression of important virulence genes [11,12], and host immune response [13,14], etc. Studies have shown that virulent strains are generally more pathogenic and invasive than attenuated strains. The *L. monocytogenes 10403s* strain in the 1/2a serotype can colonize the spleen and liver of mice in large quantities and invade Caco2 cells and replicate intracellularly [15]. Moreover, strains of serotype 4a can cause infections but cannot establish long-term infections in macrophages [16]. Compared with the 1/2a serotype strains, the 4a non-toxic strains have a shorter propagation time in the cells, but they all express similar metabolic-related proteins.

The majority of studies on *L. monocytogenes* infection have been conducted in animals such as mice and single-cell line models such as colon adenocarcinoma cells or macrophage cells [17,18]. Most of our knowledge of the innate and adaptive immune responses has been learned from experimental *L. monocytogenes* infections of mice [19]. In addition to immune cells, intestinal epithelial cells are also involved in the defense against *L. monocytogenes* infections. Studies explored that host responses caused by *L. monocytogenes* included innate immune responses first activated by the PRRs of intestinal epithelial cells, immune cells recruited by downstream cytokines, and the adaptive immune responses subsequently stimulated [19,20]. Recently, intestinal organoids have emerged as more effective infection models that reproduce the differentiation of intestinal epithelial cells and show a greatest similarity to the intestinal epithelium with respect to cell composition and structure [21]. It has been verified that organoids can be used to study the interactions between pathogens and host cells at the intestinal interface [22,23]. Therefore, the intestinal organoid is a suitable *L. monocytogenes* infection model for exploring the host response of non-immune cells.

Proteomics can provide protein information related to the biological metabolism and infection mechanisms of the host or microorganism, which may be useful to further understand the interaction between the pathogenic microorganism and the host [24]. Recently, tandem mass tag (TMT)-based proteomic platforms have been used as particularly robust proteomic techniques due to high sensitivity [25]. It has been shown that genomis and proteomic techniques can be widely used to analyze the differences of strains in transcription and protein expression [26,27,28]. Studies have shown that *L. monocytogenes* infection may have a major impact on host transcription and translation, cytoskeleton and connection, mitochondrial fission, host immune response, and the apoptosis pathway [29]. However, research on intestinal epithelial host responses caused by *L. monocytogenes* infection excluding the effects of immune cells is not yet comprehensive. Therefore, information on proteome changes in the infected intestinal organoids is necessary to understand host responses to non-immune cells.

In this study, mice and intestinal organoids were infected by two strains of *L. monocytogenes* (serotypes 1/2a and 4a), and the significant changes in the global protein expressions of the infected intestinal organoids were described by a highly sensitive quantitative approach including tandem mass tag labeling and an LC-MS/MS platform combined with advanced bioinformatics analysis in order to provide more information on the mechanisms of *L. monocytogenes* infection in the intestine.

## 2. Results

### 2.1. L. Monocytogenes Infection and Clinical Signs

**Figure 1:**
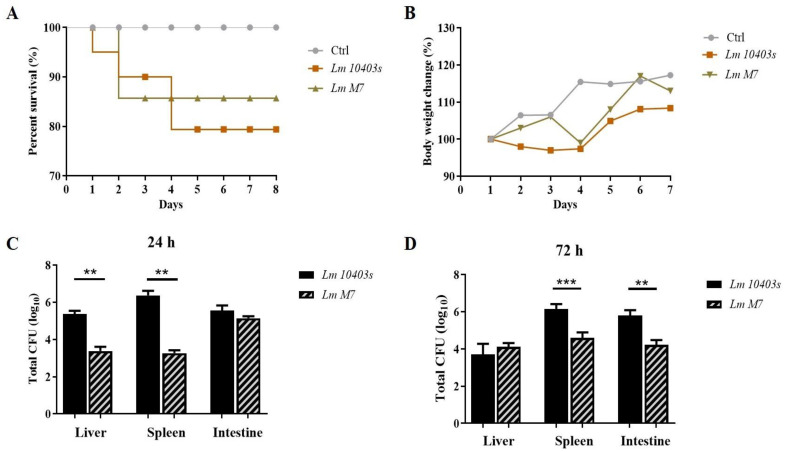
*L. monocytogenes* infection in mice. (**A**) Time course of survival rate of mice. (**B**) Time course of body weight change in mice. (**C**,**D**) Bacterial load after 24 h and 72 h of infection. ** *p* < 0.01, *** *p* < 0.001.

After randomized intragastric administration, compared with the group of low virulence strain *L. monocytogenes M7*, mice infected with *L. monocytogenes 10403s* showed higher weight loss and lower survival rates (Figure 1A). After infection for 24 h and 72 h, the number of *L. monocytogenes 10403s* cells in the intestine, liver, and spleen were higher than that of *L. monocytogenes M7* (Figure 1B). These results showed that the highly toxic *L. monocytogenes 10403s* strain resulted in more serious infections and lower survival rates.

### 2.2. Quality Validation of the Proteomic Data

**Figure 2:**
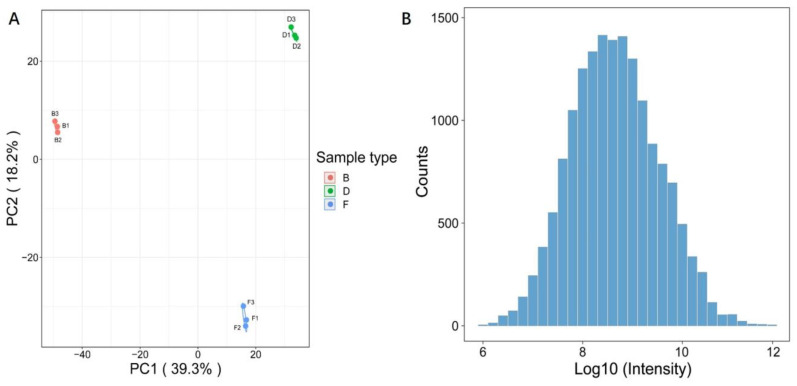
Experimental strategy for quantitative proteome analysis and quality validation. (**A**) PCA analysis of protein quantitation among each sample; (**B**) histogram of proteomics data. B—Control; D—*L. monocytogenes 10403s*; F—*L. monocytogenes M7*.

To reveal the changes in the protein levels of *L. monocytogenes* infections with different toxicity, an integrated proteomic approach was performed using organoids (one control and two infected groups). The label efficiency of each group was 99.80%, 99.60% and 99.61%, respectively, with more than 95 % label efficiency. Altogether, 6564 proteins were identified, of which 5591 proteins were quantified (Table S1). As shown in Figure 2A, the results of the principal component analysis (PCA) indicated that good repeatabilities were displayed in each sample, which was consistent with the result of the scatter plot (Figure S1). In addition, the histogram of proteomics data demonstrated that the distribution of data conformed to the normal distribution (Figure 2B).

### 2.3. Analysis of the DEPs in Organoids Infected by L. monocytogenes 10403s and M7 Infections

**Figure 3:**
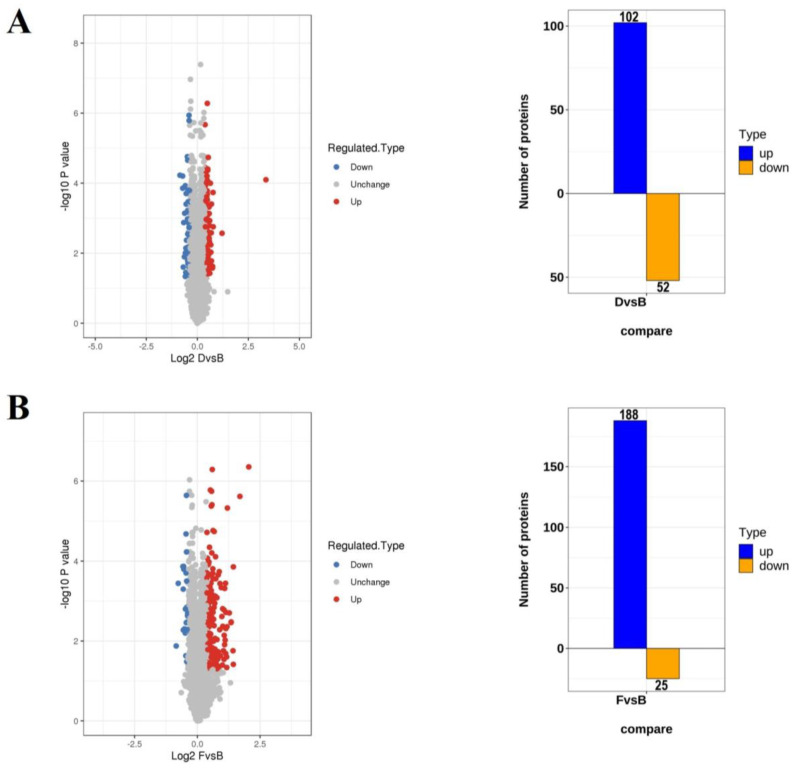

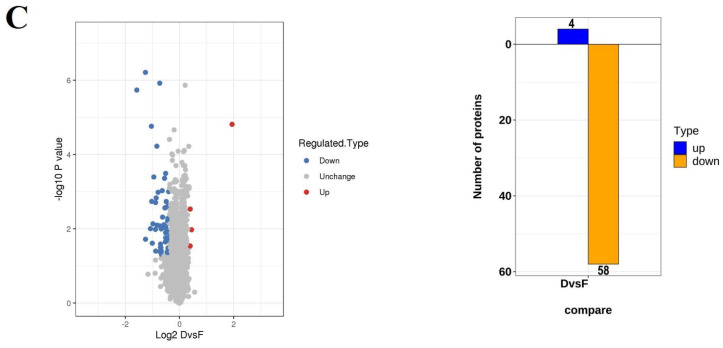
The numbers of DEPs in different comparisons. (**A**) The volcano and numbers of the up-and down-regulated proteins in *L. monocytogenes 10403s* vs. the control (D/B). (**B**) The volcano map and numbers of the up- and down-regulated proteins in *L. monocytogenes M7* vs. the control (F/B). (**C**) The volcano map and numbers of the up- and down-regulated proteins in *L. monocytogenes 10403s* vs. *L. monocytogenes M7* (D/F).

For the differentially expressed proteins (DEPs), the cutoff criteria was set with a *p* value < 0.05 and the infection vs. control group ratio at >1.3-fold difference. Among the quantitative proteins, 102 up-regulated and 52 down-regulated proteins were identified under *L. monocytogenes 10403s* infection (Figure 3A), while 188 up-regulated and 25 down-regulated proteins were identified under *L. monocytogenes M7* infection (Figure 3B). The differentially expressed proteins between *L. monocytogenes 10403s* and *L. monocytogenes M7* infection were also calculated, resulting in 4 up-regulated and 58 down-regulated proteins (Figure 3C). Table S2 presents the relative expression of DEPs in *L. monocytogenes 10403s* vs. the control (D/B) and *L. monocytogenes M7* vs. the control (F/B). The protein ratio was expressed as *L. monocytogenes* infection vs. the control.

### 2.4. Analysis of the DEPs in Organoids Infected by L. monocytogenes 10403s and M7 Infections

To further understand the DEPs in *L. monocytogenes*-infected intestinal organoids, functional classification was performed from the gene ontology (GO) analysis and subcellular structure localization in order toexplain the biological role of proteins from different angles.

**Figure 4:**
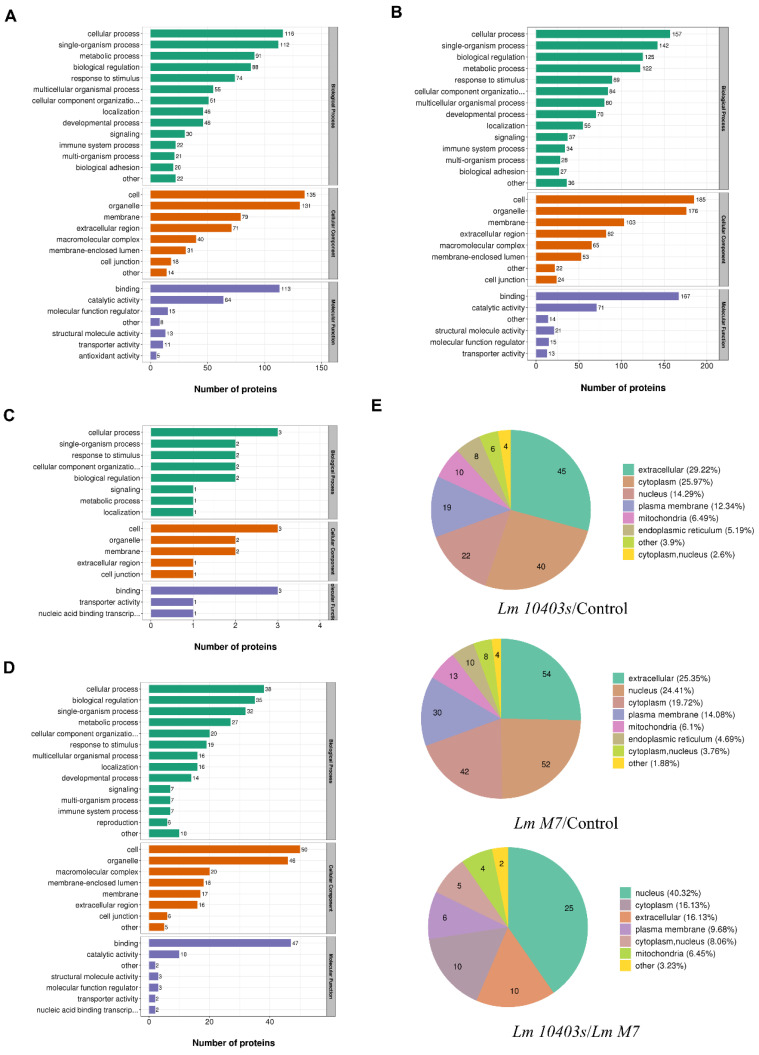
GO analysis and subcellular locations of DEPs in different comparisons. (**A**) GO analysis of regulated DEPs in Lm *10403s*/control. (**B**) GO analysis of regulated DEPs in *Lm M7*/control. (**C**) GO analysis of the up-regulated DEPs in *Lm 10403s*/*Lm M7*. (**D**) GO analysis of the down-regulated DEPs in *Lm 10403s*/*Lm M7*. All proteins were classified by GO terms. X-axis—number of DEPs. (**E**) Subcellular locations of the DEPs in different comparisons.

In the *L. monocytogenes 10403s* infection, the main biological processes of DEPs comprised the single-organism process, biological regulation and metabolism, and the defense response from stimulation and immunity. The cell composition mainly comprised the membrane and macromolecular complex, and molecular function mainly comprised binding and catalytic activity (Figure 4A). Under *L. monocytogenes M7* infection, the main biological processes of DEPs were the same as those of *L. monocytogenes 10403s*. The main conclusions of cell composition and molecular function were also similar to those of *L. monocytogenes 10403s* (Figure 4B). In comparison, between *L. monocytogenes 10403s* and *L. monocytogenes M7*, up-regulated proteins were grouped into single-organism processes, membranes and bindings (Figure 4C), while down-regulated proteins were related to biological regulation and metabolism, immune system processes, the macromolecular complex and catalytic activity (Figure 4D).

In the subcellular localization of DEPs, the host cell proteins infected by *L. monocytogenes 10403s* were mainly distributed in the extracellular region and the cytoplasm, and the proteins of *L. monocytogenes M7* infection were mainly distributed in the extracellular region and the nucleus. Comparing both infections, the DEPs were mainly distributed in the nucleus (Figure 4E).

### 2.5. Enrichment Analysis of the DEPs in Organoids Infected by L. monocytogenes 10403s and M7

**Figure 5:**
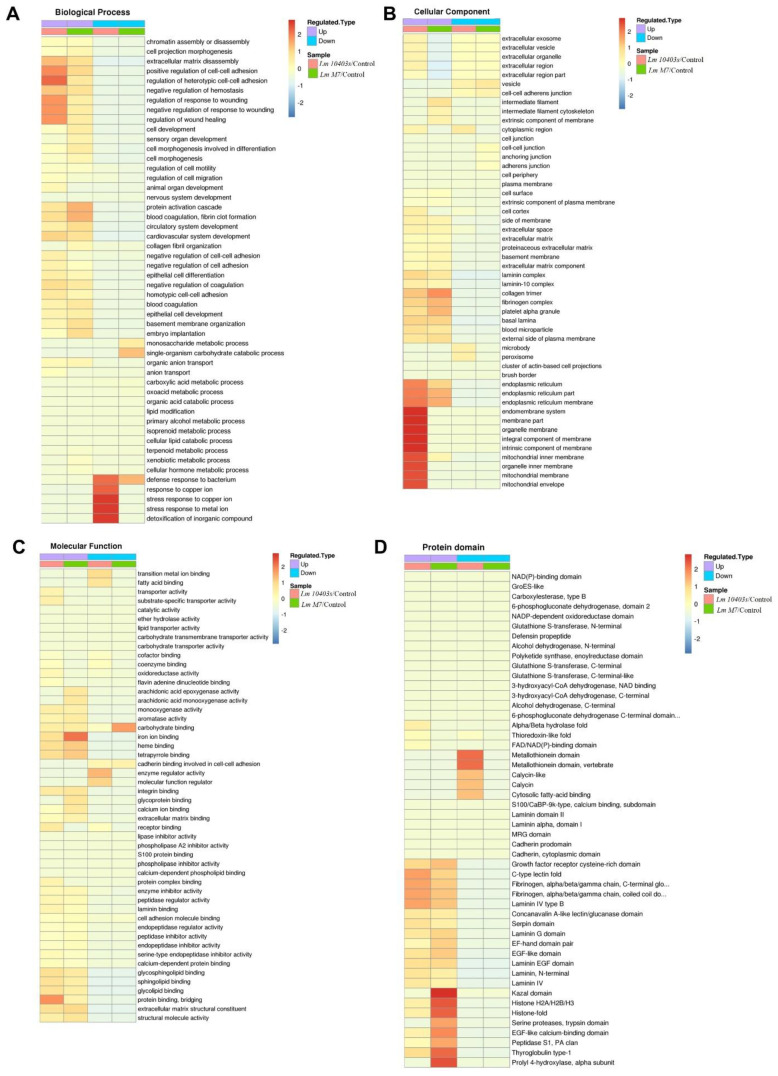
GO and protein domain enrichment analysis of the DEPs. Heatmaps show the enrichments of the DEPs in different comparisons with GO annotation belonging to biological processes (**A**), cellular components (**B**), and molecular function (**C**). (**D**) Significantly enriched protein domains of the DEPs.

To find out whether the DEPs had a significant enrichment trend in certain functional types, an enrichment analysis of GO classification and protein domain were performed in each comparison group (Figure 5).

Under *L. monocytogenes 10403s* infection, the significantly enriched biological processes were mainly associated with defense responses to bacteria, the antimicrobial humoral response, and extracellular matrix disassembly. The significantly enriched cellular component was related to the extracellular matrix and the basement membrane, while molecular function mainly correlated with the extracellular matrix structural constituent and oxidoreductase activity and various bindings, such as the glycosphingolipid binding. In addition, the significantly enriched domain terms were the laminin EGF domain, laminin and fibrinogen, and metallothionein.

In *L. monocytogenes M7* infection, the significantly enriched biological processes were mainly associated with protein activation cascades, antimicrobial humoral response, and extracellular matrix disassembly. The significantly enriched cellular components were basically the same as *L. monocytogenes 10403s,* while molecular function primarily correlated with the extracellular matrix structural constituent, iron ion binding, and structural molecule activity. Nonetheless, the top three significantly enriched domain terms were the same as *L. monocytogenes 10403s*, while the remaining domains included histone.

For both *L. monocytogenes 10403s* and *L. monocytogenes M7*, the significantly enriched biological processes were mainly associated with a negative regulation of gene expression, biosynthetic processes, and cellular macromolecular complex assembly, whereas the cellular components were related to nucleosomes and the DNA packaging complex. The significantly enriched molecular functions were essentially linked to chromatin DNA binding and nucleosomal DNA binding. The domain terms were histone and histone-fold.

### 2.6. KEGG Analysis of the DEPs in Organoids Infected by L. monocytogenes 10403s and M7

**Figure 6:**
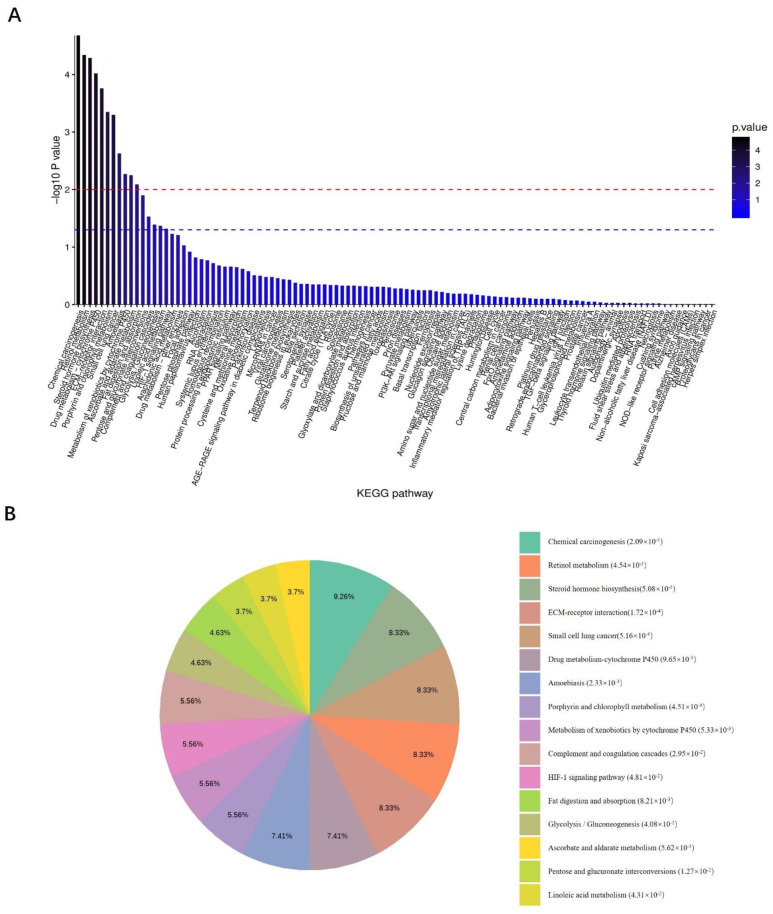
KEGG enrichment analysis of all DEPs obtained from the combination of the *Lm 10403s* infection group and the *Lm M7* infection group. All differentially expressed proteins in the *Lm* *10403s* infection group and the *Lm M7* infection group were put together for KEGG analysis. (**A**) Columnar section of the KEGG enrichment analysis results. (**B**) Pie chart of the KEGG enrichment analysis results.

All DEPs in the *L. monocytogenes 10403s* and *M7* infection groups were put together for KEGG analysis. Figure 6A demonstrates that all the KEGG pathways were enriched. The most significant enrichment was chemical carcinogenesis, which reflected the genotoxic and non-genotoxic effects of *L. monocytogenes* on the host. The main significant enrichments were metabolic-related pathways, such as retinol metabolism, steroid hormone biosynthesis, and the drug metabolism cytochrome P450. Likewise, fat digestion and absorption, glycolysis/gluconeogenesis and other metabolic pathways were also enriched.

Notably, the DEPs reflecting the ability of *L. monocytogenes* to cause damage during the invasion were significantly involved in small cell lung cancer, amoebiasis, and some other host responses, such as the ECM–receptor interaction, complement and coagulation cascades, and the HIF-1 signaling pathway. Moreover, it can be seen from Figure 6B that some signaling pathways related to host defense and immune response were enriched, such as apoptosis and autophagy, ferroptosis, the NOD-like receptor signaling pathway, etc. (Figure 6B).

**Figure 7:**
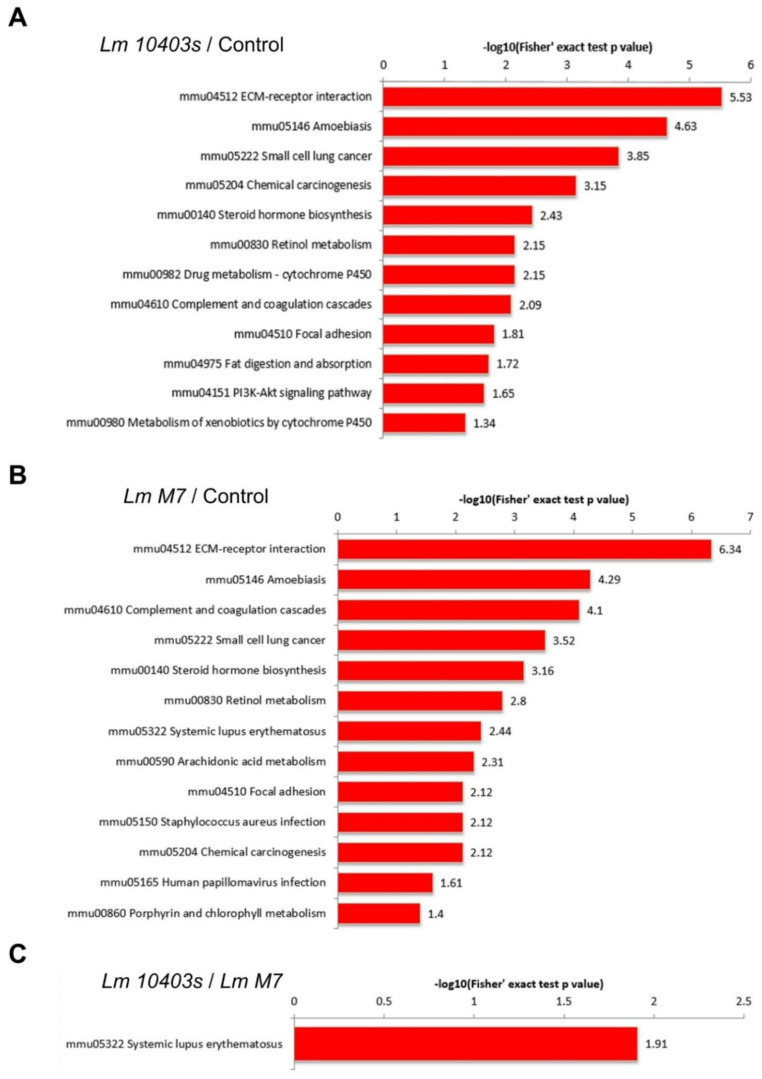
KEGG enrichment analysis of the DEPs. (**A**) Significantly enriched KEGG terms of the DEPs in the *Lm 10403s* vs. control comparison. (**B**) Significantly enriched KEGG terms of the DEPs in the *Lm M7* vs. control comparison. (**C**) Significantly enriched KEGG terms of the DEPs in the *Lm 10403s* vs. *Lm M7* comparison.

Figure 7 illustrates that the KEGG pathway of the *L. monocytogenes 10403s* and *M7* infection groups were mainly related to invasion, host response, and metabolism. Among the KEGG pathways in which up-regulated proteins were enriched in the *L. monocytogenes 10403s* infection group were the pathways related to bacterial adhesion and invasion including amoebiasis, small cell lung cancer, chemical carcinogenesis, and focal adhesion. Some host responses were also included, such as ECM–receptor interactions, complement and coagulation cascades, and the PI3K-Akt signaling pathway. Moreover, the KEGG pathways related to metabolic processes contained steroid hormone biosynthesis, retinol metabolism, and drug metabolism cytochrome P450.

Similar results were found in the KEGG pathway enriched in the *L. monocytogenes M7* infection group. Up-regulated proteins were enriched in the same three pathways related to bacterial adhesion and invasion as *L. monocytogenes 10403s*, were involved in ECM–receptor interactions and complement and coagulation cascades, and were enriched in three host lipid metabolism-related metabolic processes. However, unlike the virulent strain *L. monocytogenes 10403s*, the up-regulated proteins of *L. monocytogenes M7* contributed to systemic lupus erythematosus, and the down-regulated proteins were only enriched in glycolysis/gluconeogenesis. As for the *L. monocytogenes 10403s* vs. *M7* group, the DEPs were enriched in two pathways: systemic lupus erythematosus and transcriptional mis-regulation in cancer.

In the intestine organoid model, the innate immune response was the primary host defense response caused by *L. monocytogenes*. Therefore, we selected the DEPs of five pathways in the *L. monocytogenes 10403s* vs. control group and the *L. monocytogenes M7* vs. control group which were related to the immune system process. Then, these proteins were accurately analyzed at 1.3-fold and 1.2-fold differential folds. The five pathways included ECM–receptor interaction, the complement and coagulation cascade, the HIF-1 signaling pathway, ferroptosis, and the NOD-like receptor signaling pathway.

Among the ECM–receptor interactions, the significantly up-regulated proteins which were consistent in the *L. monocytogenes 10403s* and *M7* groups were *Fn1*, *Lamc1*, *Lama1*, *Lamb1*, *Col4a2*, *Col4a1*, *Lamb2*, *Hspg2*, and *Agrn*. Moreover, *Agrn* was up-regulated but not significant in the *L. monocytogenes 10403s* group, while 1.3-fold significantly up-regulated in the *L. monocytogenes M7* group, and 1.2-fold significantly up-regulated in the *L. monocytogenes 10403s* vs. *M7* comparison group. This showed that *L. monocytogenes M7* could significantly increase *Agrn* 1.3-fold, and the degree of up-regulation was 1.2-fold more significant than *L. monocytogenes 10403s*.

For the complement and coagulation cascades, six differential proteins were identified in both the *L. monocytogenes 10403s* and *M7* infection groups: *Plg*, *Fgb*, *Fga, Fgg*, *Clu*, and *C3*. These differential proteins were significantly up-regulated in the *L. monocytogenes 10403s* and *M7* infection groups, with no marked change in the comparison group. Moreover, *F10* significantly varied in the *L. monocytogenes 10403s* vs. *M7* comparison group, being significantly up-regulated (1.3-fold) in the latter and down-regulated but not significantly in the former group. This showed that the effect of *L. monocytogenes M7* on *F10* was opposite to *L. monocytogenes 10403s*, and the degree of activation was significantly higher than *L. monocytogenes 10403s*.

In the NOD-like receptor signaling pathway, the differential proteins shared by the *L. monocytogenes 10403s* and *M7* infection groups were significantly down-regulated. Among them, *Nod2* and *Pycard* were significantly (1.3-fold) reduced in both the *L. monocytogenes 10403s* and *M7* groups, while a 1.2-fold marked reduction was observed in *Nampt*. Particularly, *Txn2* and *Nek7* were significantly down-regulated in the *L. monocytogenes 10403s* vs. *M7* comparison group. Although *L. monocytogenes 10403s* was down-regulated and *M7* was up-regulated, there was no significant change. This indicated that the activation of *L. monocytogenes M7* on these proteins was significantly different from *L. monocytogenes 10403s*.

In the HIF-1 signaling pathway, the significant differentially up-regulated proteins shared by the *L. monocytogenes 10403s* and *M7* infection groups were *Tfrc*, *Tf, Hkdc1* and *Cdkn1b*, and the significantly down-regulated protein was *Eno1*. However, only *Slc2a1,* which increased, differed markedly in the *L. monocytogenes 10403s* vs. *M7* comparison group.

For ferroptosis, the significantly up-regulated proteins shared by the *L. monocytogenes 10403s* and *M7* infection groups were *Acsl5*, *Tf, Acsl1*, *Tfrc*, and *Lpcat3*, and the significantly down-regulated proteins were *Fth1* and *Ftl1*: neither changed significantly in the comparison group. In addition, *L. monocytogenes 10403s* significantly increased by 1.2-fold *Gpx4*, *Vdac3* and *Vdac2*, which were up-regulated but not significant in the *L. monocytogenes M7* group.

### 2.7. Confirmation of Proteomic Data by RT-PCR

**Figure 8:**
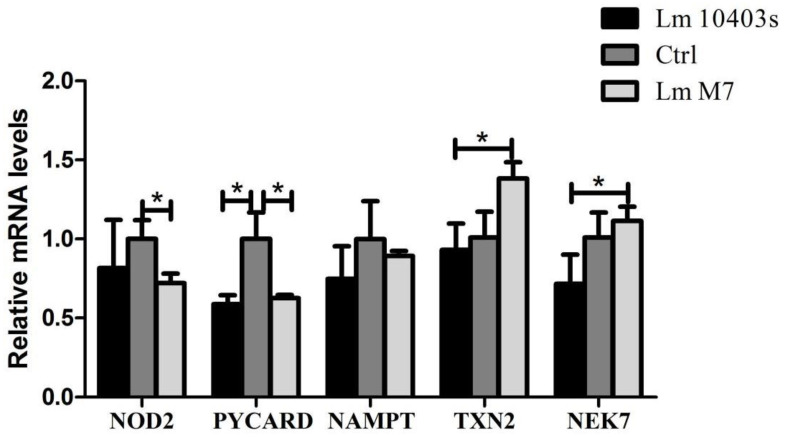
Expression levels of the DEPs in NOD-like receptor-signaling pathway were verified using RT-PCR. * *p* < 0.05.

Among the five related pathways, only the differential proteins in the NOD-like receptor signaling pathway were down-regulated. This showed that the effect of *L. monocytogenes* infection on the NOD-like receptor signaling pathway was mainly to inhibit or reduce the differential proteins. The results highlighted that *L. monocytogenes* strains with different toxicity significantly decreased the mRNA expression level of *NOD2* (Figure 8). However, in terms of the other genes in the NOD pathway, *L. monocytogenes 10403s* significantly down-regulated *Pycard* and *Nampt*. Compared to the *L. monocytogenes 10403s* group, the relative mRNA levels of *Txn2* and *NEK7* were significantly increased in the *L. monocytogenes M7* group, which corroborated the KEGG analysis of the DEPs in the NOD signaling pathway.

## 3. Discussion

Listeriosis is one of the most serious food-borne diseases and is mainly caused by contaminated food. *L. monocytogenes* can enter the digestive tract and then cross the intestinal barrier, which is a critical step in systemic infection [30]. Research on the stage of intestinal infection has mostly analyzed the virulence genes and survival mechanisms of *L. monocytogenes* in addition to host response [31,32]. In the proteomics analysis of *L. monocytogenes*, many studies have focused on bacterial proteins and explored the relationship between some proteins and bacterial virulence by comparing the different protein expressions between different strains in terms of stress, biological metabolism, and virulence genes [16,26]. Others have focused on host protein change, and investigated the interaction between host response and bacterial virulence [33]. However, studies on changes in intestinal epithelial host proteins are not comprehensive.

In the present study, TMT-based quantitative proteomic analysis was used to compare the total proteomes in organoids infected by different toxic *L. monocytogenes* strains. Quantitative analysis demonstrated 154 differentially expressed proteins in the virulent-strain-infected organoids, 213 proteins in the low-virulence-strain-infected organoids, and 62 proteins in the *L. monocytogenes 10403s* vs. *L. monocytogenes M7* comparison group. These proteins were found to be involved in cell transport, binding, biological metabolism, energy metabolism, transcriptional regulation, signal transduction, and defense response.

The infection of *L. monocytogenes* in intestinal organoids is a process that involves many proteins and pathways. The results of analyzing the *L. monocytogenes 10403s* and *M7* groups showed that the damage of *L. monocytogenes* infection was mainly reflected in the destruction of the intestinal barrier, affecting the disease-signaling pathway and changing the metabolic process of the host cells. Additionally, different toxic *L. monocytogenes* strains led to changes in five important pathways related to the host immune process.

ECM–receptor interaction is a micro-environmental pathway that maintains cell and tissue structure and function, leading to the direct or indirect control of cellular activities such as adhesion, migration, differentiation, proliferation, and apoptosis. Recent studies have identified that this pathway is possibly involved in the development of breast cancer [34]. Studies confirm that these proteins are utilized by pathogens to adhere to and invade host tissues, and can increase the adherence of *L. monocytogenes* to HEp-2 cells [35,36]. *L. monocytogenes* up-regulated many ECMs during the infection, such as fibronectin, laminin, and collagen, indicating that *L. monocytogenes* can improve the adhesion and invasion of cells by adjusting ECMs.

The significantly different proteins in the hypoxia-inducible factor-1 (HIF-1) signaling pathway and ferroptosis were *Tfrc* and *Tf*, which regulated intracellular iron. They were both up-regulated in the *L. monocytogenes 10403s* and *M7* infection groups. It was found that transferrin (*Tf)*, the transferrin receptor (*Tfrc*), and ferroportin favored oxidative damage and ferroptosis by increasing iron uptake and reducing iron export [37]. However, HIF-1 was not identified, which may be caused by the lack of immune cells in small intestine organoids. Nevertheless, there was no significant variation in the key regulatory protein glutathione peroxidase 4 (Gpx4) in the ferroptosis pathway, indicating that the effect of different toxic *L. monocytogenes* on ferroptosis was not critical.

The complement, coagulation, and fibrinolytic systems can form a serine protease system, playing essential roles in innate immune responses. The interplay between complement and coagulation contributes to strengthening innate immunity and activates adaptive immunity to eliminate bacteria [38]. However, the dysfunction of the complement system and central fractional coupling activity or inhibition of the coagulation cascade could lead to serious diseases, such as sepsis and systemic lupus erythematosus [38]. In this study, three fibrinogen chains (*Fga*, *Fgb* and *Fgg*) were up-regulated in two *L. monocytogenes* infections, while coagulation factor X (*F10*) and *C3* were up-regulated only in the *L. monocytogenes M7* infection group. As for the *L. monocytogenes 10403s* vs. *L. monocytogenes M7* comparison group, the DEPs were significantly enriched in systemic lupus erythematosus. This indicated that the different toxic *L. monocytogenes* activated the complement system and coagulation cascades in the stage of intestinal infection, and low-virulence strains caused a more significant coagulation cascade.

The NOD-like receptor signaling pathway is mediated by NOD-like receptors in the host cell and is also an important innate immune response [39]. *NOD2* can be expressed in intestinal epithelial cells, such as Paneth and stem cells [40]. Moreover, extensive studies have shown that *NOD2* plays an important role in maintaining the balance between bacteria, epithelial cells, and the innate immune response of the host [41]. *NOD2* recruits downstream proteins by recognizing the muramyl dipeptide (MDP) in the cell wall of pathogens, and then induces the activation of the NF-κB, MAPK, and caspase-1 pathways [42]. Bacteria will activate *NOD2* during infection and increase the expression level of *NOD2*. Interestingly, *NOD2* was down-regulated in both the *L. monocytogenes* 10403s and *L. monocytogenes M7* groups.

Studies have shown that, under the stimulation of different concentrations of MDP, *NOD2* is activated in dental pulp stem cells, but the expression level is reduced [43]. This suggests that *NOD2* in stem cells could be inhibited by MDP. More so, the expression of *NOD2* in the terminal ileum of sterile mice has been observed to be low, with its expression increasing after supplementation with symbiotic bacteria [44]. This indicates that the expression level of *NOD2* in the intestine is related to commensal bacteria. Therefore, the reason for the low expression of *NOD2* in the small intestine organoid model could be related to the lack of symbiotic bacteria.

Furthermore, the formation of inflammatory corpuscle complexes and the activation of caspase-1 occurs downstream of the NOD-like receptor-signaling pathway. *Pycard* (also known as ASC) is a key adaptor protein of inflammatory bodies (such as NLRP3) and an essential protein that activates inflammatory responses and apoptosis signaling pathways [45]. In the *L. monocytogenes 10403s* and *M7* groups, the expression of *Pycard* was down-regulated, indicating that *L. monocytogenes* may down-regulate *Pycard* to inhibit the formation of inflammatory corpuscle complexes. Additionally, in both groups, *Txn2* and *Nek7* related to NLRP3 inflammatory body assembly and were significantly down-regulated. Specifically, they were down-regulated by *L. monocytogenes 10403s* and up-regulated by *L. monocytogenes M7*, while their change difference did not reach 1.2 times. This demonstrates that the variation between the two strains in the NOD-like receptor-signaling pathway in the host is mainly found in the regulation of inflammasome assembly.

Overall, KEGG enrichment analysis showed that different toxic *L. monocytogenes* increased the expression of adhesion and invasion-related proteins, reduced the energy metabolism of host, and triggered various host defense responses. Moreover, the virulent strain *L. monocytogenes 10403s* had a more significant activation effect on the ferroptosis pathway, while the low virulent strain *L. monocytogenes M7* has a more significant activation effect on the complement system. More importantly, *L. monocytogenes* strains with different toxicity could affect the proliferation and cell protection of intestinal stem cells by down-regulating *NOD2*. The down-regulated proteins of the *L. monocytogenes 10403s* vs. *M7* comparison group were enriched in systemic lupus erythematosus and in transcriptional mis-regulation in cancer. This suggests that the low virulent strain had a stronger interference effect on immunodeficiency disease and transcriptional regulation.

In summary, complex responses to virulent and low virulent *L. monocytogenes* infections were revealed by TMT-based quantitative proteomics analysis using intestinal organoids. The DEPs of the *L. monocytogenes 10403s-* and *M7*-infected groups displayed similar biological functions and subcellular localizations to previous analysis. The difference in their influence on host biological function was mainly reflected in transcription regulation and metabolism. These different DEPs were mainly distributed in the nucleus, and their domains were related to histones. Furthermore, complement and coagulation cascade and the NOD-like receptor-signaling pathway were detected as the innate immune responses caused by the two strains. These results reveal the modulation of protein expression by *L. monocytogenes* to overcome host defense responses, and the data may give a comprehensive resource for investigating the overall responses of intestinal epithelial cells, excluding immune cells, to infection with different toxic *L. monocytogenes* strains.

## 4. Materials and Methods

### 4.1. Bacterial Strains, Animals, and Intestinal Organoids

The *L. monocytogenes 10403s* (1/2a serotype strain) and *M7* (4a serotype strain) strains, which were erythromycin resistant, were obtained from Prof. Weihuan Fang (Zhejiang University, Hangzhou, China). The *L. monocytogenes* samples were grown in brain–heart infusion (BHI) broths supplemented with 5 µg/mL erythromycin at 37 °C for 16 h.

Intestinal organoids were cultured from the intestines of 4-week-old C57BL/6 mice (Animal Research Centre of Yang Zhou University, Yangzhou, China). The intestine was flushed out with phosphate-buffered saline (PBS) and was cut into small pieces. Next, tissues were rocked in DPBS containing 2 mM EDTA for 30 min at 4 °C. After incubation, crypts were released by vigorous shaking, and cells were filtered through a 70-μm sterile cell strainer. Then, crypts were collected by centrifugation at 700 rpm for 5 min, mixed with Matrigel (Corning, NY, USA), and seeded into a 24-well tissue culture plate. The plate was incubated for 15 min at 37 °C to polymerize. Finally, complete crypt culture medium was added to each well containing advanced DMEM/F12 supplemented with penicillin–streptomycin, 10 mM HEPES, 2 mM glutamine, N2, B27 (Gibco, NY, CA, USA; Life Technologies, Carlsbad, CA, USA), EGF (50 ng/mL, Peprotech, Cranbery, NY, USA), R-spondin1 (500 ng/mL, Peprotech, Cranbery, NY, USA), Noggin (100 ng/mL, Peprotech, Cranbery, NY, USA), and Y-27632 (10 mM, Sigma, AL, USA). The medium was changed every 2–3 days, and organoids were passaged every 3–5 days.

### 4.2. Experimental Design and L. monocytogenes Infection

Thirty C57BL/6 mice (4 weeks old; specific-pathogen-free (SPF) females) were randomly divided into three groups and orally administrated sterile PBS (control group, CK), *L. monocytogenes* *10403s* (10^9^ CFU/mL, *LM 10403s*) and *L. monocytogenes M7* (10^9^ CFU/mL, *LM* *M7*). At 24, 72, and 96 h after infection, the mice were sacrificed and CFUs in the intestine, liver and spleen were determined by dilution coating on BHI plates with 5 µg/mL erythromycin and PALCAM agar plates. The body weight and survival rate were recorded for a week after inoculation. All animal studies were approved by Nanjing Agriculture University Committee on Animal Resources Committee and the National Institutes of Health guidelines for the performance of animal experiments (Approval ID: NJAU.No20210305009).

Organoids were cultured in complete crypt culture medium at 37 °C in a 5% CO_2_ atmosphere and infected with 1 × 10^7^ CFU of the virulent strain *L. monocytogenes 10403s*, or 1 × 10^7^ CFU of the attenuated strain *L. monocytogenes M7* and culture medium for 1 h. After infection, organoids were centrifuged at 900 rpm for 5 min, and extracellular bacteria were removed by washing twice with DPBS. Finally, organoids were embedded into fresh Matrigel (Corning, NY, USA) and cultured for 18 h, and the media were refreshed with penicillin–streptomycin media for the experiment.

### 4.3. Protein Sample Preparation

Organoids were infected in three groups (virulent strain infection (group D), attenuated strain infection (group F), and control (group B)) at 18 h after culture, with three replicate samples in each group. Samples were sonicated three times on ice using a high-intensity ultrasonic processor (Scientz, Hangzhou, China) in lysis buffer (8 M urea, 1% Protease Inhibitor Cocktail). The remaining debris was removed by centrifugation at 12,000× *g* for 10 min. Finally, the supernatant was collected and the protein concentration was determined with a BCA kit according to the manufacturer’s instructions.

### 4.4. Trypsin Digestion

For trypsin digestion, the equal protein solution of each group was reduced with 5 mM dithiothreitol for 30 min at 56 °C, and alkylated with 11 mM iodoacetamide for 15 min at room temperature in darkness. The protein sample was then diluted by adding 100 mM TEAB to a urea concentration of less than 2M. Finally, trypsin was added at a 1:50 trypsin-to-protein mass ratio for the first digestion overnight and a 1:100 trypsin-to-protein mass ratio for a second 4 h digestion. Approximately 100 μg of protein for each sample was digested with trypsin for the following experiments.

### 4.5. TMT Labeling

After trypsin digestion, peptide was desalted with a Strata X C18 SPE column (Phenomenex (Sigma, AL, USA)) and vacuum-dried. The same amount of peptide was reconstituted in 0.5 M TEAB and processed according to the manufacturer’s protocol for TMT kit. Briefly, one unit of TMT reagent (defined as the amount of reagent required to label 100 μg of protein) was thawed and reconstituted in acetonitrile. The peptide mixtures were then incubated for 2 h at room temperature and pooled, desalted, and dried by vacuum centrifugation. Prepared samples were stored at −80 °C until liquid chromatography–mass spectrometry (LC–MS/MS) analysis.

### 4.6. HPLC Fractionation

The samples were fragmented into a series of fractions by high-pH reverse-phase HPLC using an Agilent 300Extend C18 column (5 μm particles, 4.6 mm ID, and 250 mm length). Briefly, peptides were first separated with a gradient of 8% to 32% acetonitrile (pH 9.0) over 60 min into 60 fractions. Then, the peptides were combined into 18 fractions and dried by vacuum centrifuging.

### 4.7. LC-MS/MS

The peptides were dissolved in liquid chromatography mobile phase A (0.1% formic acid and 2% acetonitrile) and separated using the EASY-nLC 1000 ultra-high-performance liquid system. Mobile phase B was an aqueous solution containing 0.1% formic acid and 90% acetonitrile. The liquid phase gradient setting was as follows: 0–2 min, 6–22% B; 42–54 min, 22–30% B; 54–57 min, 30–80% B; 57–60 min, 80% B, all at a constant flow rate of 500 nL/min.

The peptides were placed into the NSI ion source for ionization, and then analyzed by Orbitrap Fusion Lumos^TM^ mass spectrometry. The ion source voltage was set to 2.4 kV, and the peptide precursor ions and their secondary fragments were detected and analyzed using high-resolution Orbitrap. The m/z scan range of full scan was 350 to 1550, and the resolution of the complete peptide detected in Orbitrap was 60,000; the scan range of the second-level mass spectrometer was fixed at 100 m/z, and the resolution was set to 15,000. The data acquisition mode used a data-dependent scanning (DDA) program. To improve the effective utilization of the mass spectrum, the automatic gain control (AGC) was set at 5E4; the signal threshold was set at 10,000 ions/s; the maximum injection time was set at 60 ms; and the dynamic exclusion time of the tandem mass spectrometry scan was set at 30 s to avoid precursor ions repeating the scan.

In addition, unique peptides were used for the quantified amounts. The relative quantitative values of each sample were calculated as follows:

The raw LC-MS datasets were first searched against the database and converted into matrices containing the reported intensity of the peptides across the samples. The relative quantitative value of each protein was then calculated based on these intensity values by the following steps:

1. Firstly, the intensities of peptides (*I*) across all samples were centralized and transformed into their values of relative quantification (*U*) in each sample. The formula is as follows: *i* denotes the sample and *j* denotes the peptide.

*U_ij_ = I_ij_/Mean(I_j_)* (1)

2. To adjust the systematic bias of the identified peptide amounts among the different samples in the process of mass spectrometry detection, the relative quantitative values of the peptides needed to be corrected by the median value as follows:

*NR_ij_ = U_ij_/Median(U_i_)* (2)

3. The relative quantitative value of a protein (*R*) was calculated by the intensity median of its corresponding unique peptides. The formula is listed as follows where *k* denotes the protein and *j* denotes the unique peptides belonging to the protein:

*R_ik_ = Median(NR_ij_, j*
*∈k)* (3)

### 4.8. Data Analysis

The resulting MS/MS data were processed using the Maxquant search engine (v.1.5.2.8). Tandem mass spectra were searched against the SwissProt Mouse database concatenated with a reverse decoy database. An anti-library was added to calculate the false discovery rate (FDR) caused by random matching, and a common contamination library was added to the database to eliminate the impact of the contaminated proteins in the identification results. Trypsin/P was specified as cleavage enzyme, allowing up to 2 missing cleavages. The minimum length of the peptide was set at 7 amino acid residues; the maximum number of modifications of the peptide was set at 5; the mass errors of the primary precursor ions of the first search and the main search were set at 20 ppm and 5 ppm, respectively; and the mass error of the secondary fragment ion was 0.02 Da.

The cysteine alkylation was set as a fixed modification, and the variable modification was the oxidation of methionine, acetylation at the N-terminus of the protein, and deamidation (NQ). The quantitative method was set at TMT-6plex FDR for protein identification, PSM identification was set at 1%, and the minimum score for peptides was set at >40. For the differentially expressed proteins (DEPs), the cutoff criteria considered was set with a *p* value < 0.05 and the infection vs. control group a ratio >1.3-fold difference.

### 4.9. Real-Time Quantitative PCR

Total RNA was extracted from the organoid samples using RNAiso Plus (Takara, Beijing, China). Then, the reverse transcription of the RNA was performed. The thermal cycling conditions were 5 min at 95 °C, followed by 40 cycles of 15 s at 95 °C and 34 s at 60 °C using an Applied Biosystems 7500 real-time PCR system, as described previously. The mRNA expression level of each target gene was normalized to the expression level of GAPDH, and the expression levels of the uninfected organoids comparing the expression levels of the infected organoids were normalized as 1, which was analyzed by ΔΔCt. All real-time PCR reactions were performed in triplicate. The primer sequences of the target and reference genes designed in NCBI are shown in Table 1.

## Figures and Tables

**Table 1:** Primer sequences used for RT-qPCR.

Target Genes	Primer Sense (5′-3′)	Primer Antisense (5′-3′)
*NOD2*	CAGGTCTCCGAGAGGGTACTG	GCTACGGATGAGCCAAATGAAG
*PYCARD*	CTTGTCAGGGGATGAACTCAAAA	GCCATACGACTCCAGATAGTAGC
*NAMPT*	GCAGAAGCCGAGTTCAACATC	TTTTCACGGCATTCAAAGTAGGA
*TXN2*	CTCGCTTGCTAGTGACTACAC	AGTGCAAACAGCGTCTCGTT
*NEK7*	GCTGTCTGCTATATGAGATGGC	CCGAATAGTGATCTGACGGGAG
*GAPDH*	ATGGTGAAGGTCGGTGTGAA	TGGAAGATGGTGATGGGCTT

## Data Availability

Not applicable.

## Supplementary Materials

The following supporting information can be downloaded at: https://www.mdpi.com/article/10.3390/ijms23116231/s1.
