# Supplementary material for: TMT-Based Quantitative Proteomic Analysis of Intestinal Organoids Infected by Listeria monocytogenes Strains with Different Virulence
Source: Int J Mol Sci. 2022 Jun 2;23(11):6231. doi: 10.3390/ijms23116231 (PMC9181811; doi:10.3390/ijms23116231)
Supplement: Supplementary file 1 [file ijms-23-06231-s001.zip › Figure S1 Scatter plots with three biological replicates among each other.pdf]

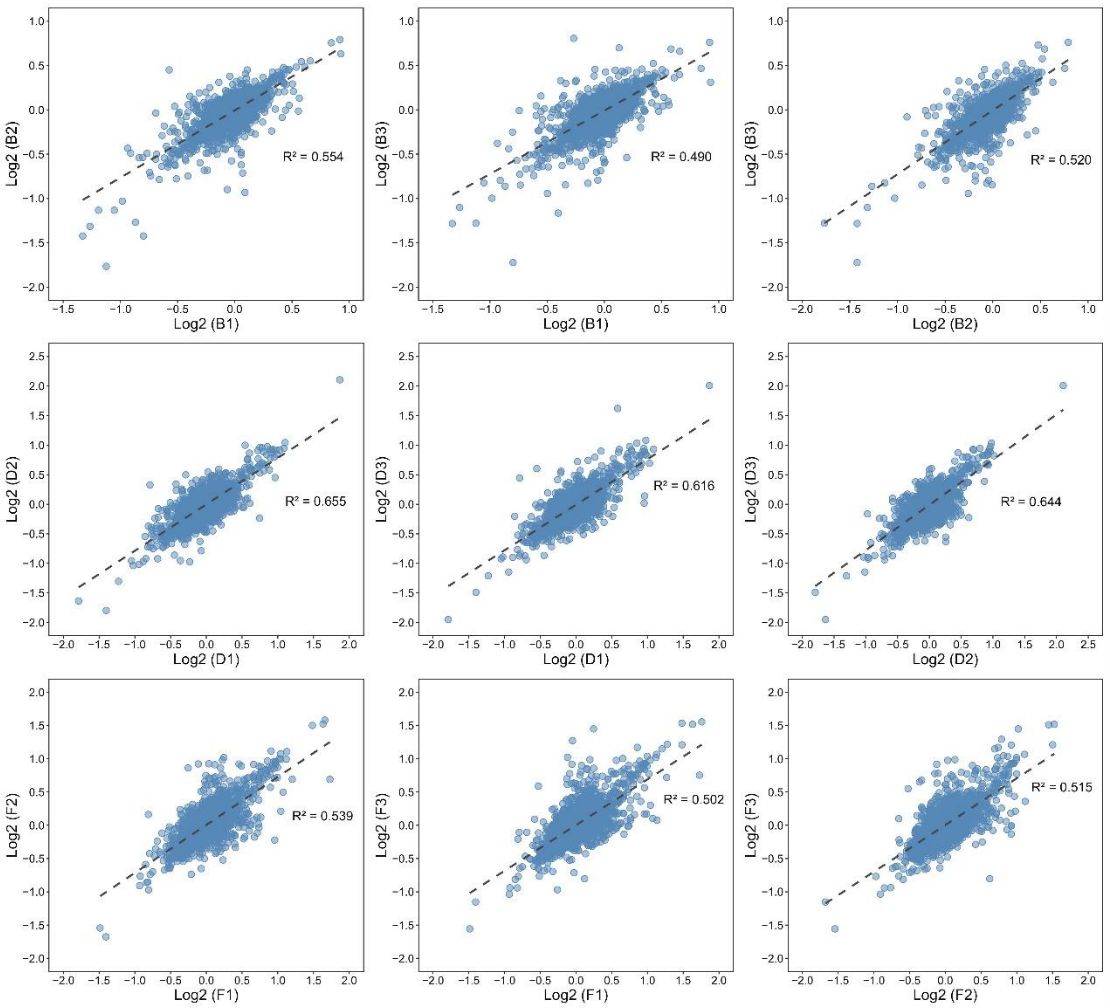

Figure S1 Scatter plots with three biological replicates among each other. B: Control; D: *L. monocytogenes 10403s*; F: *L. monocytogenes M7*.
