# Supplementary material for: TMT-Based Quantitative Proteomic Analysis of Intestinal Organoids Infected by Listeria monocytogenes Strains with Different Virulence
Source: Int J Mol Sci. 2022 Jun 2;23(11):6231. doi: 10.3390/ijms23116231 (PMC9181811; doi:10.3390/ijms23116231)
Supplement: Supplementary file 1 [file ijms-23-06231-s001.zip › Table S2 Relative expression of DEPs in Lm 10403s vs. Control and Lm M7 vs. Control.pdf]

Table S2 Relative expression of DEPs in *Lm 10403s* vs. Control (D/B) and *Lm M7* vs. Control (F/B)

Protein accession	Protein description	Gene name	MW [kDa]	D/B Ratio	F/B Ratio
A2A6A1	G patch domain-containing protein 8	Gpatch8	164.99	1.068	1.318
A2AFS3	UPF0577 protein KIAA1324	Kiaa1324	110.68	1.675	2.123
A2ASQ1	Agrin	Agrn	207.54	1.133	1.36
A4Q9F1	Protein monoglycylase TTLL8	Ttll8	94.914	0.65	0.59
A6BLY7	"Keratin, type I cytoskeletal	Krt28	50.346	1.137	1.457
B2RWS6	Histone acetyltransferase p300	Ep300	263.3	1.165	1.409
E9PV24	Fibrinogen alpha chain	Fga	87.428	1.404	1.492
E9Q414	Apolipoprotein B-100	Apob	509.43	1.302	1.204
E9Q4F7	Ankyrin repeat domain-containing protein 11	Ankrd11	296.18	1.451	1.415
F2YMG0	Serine protease 56	Prss56	65.133	1.136	2.246
O08739	AMP deaminase 3	Ampd3	88.651	1.227	1.304
O08811	General transcription and DNA repair factor IIH helicase subunit XPD	Ercc2	86.841	1.182	1.395
O35054	Claudin-4	Cldn4	22.338	2.314	2.539
O35215	D-dopachrome decarboxylase	Ddt	13.077	0.744	0.859
O35682	Myeloid-associated differentiation marker OS=Mus musculus	Myadm	35.284	1.354	1.17
O35685	Nuclear migration protein nudC	Nudc	38.358	0.765	0.789
O54750	Cytochrome P450 2J6	Cyp2j6	57.791	1.278	1.354
O55071	Cytochrome P450 2B19	Cyp2b19	55.996	1.309	1.532
O70303	Cell death activator CIDE-B	Cideb	24.8	1.398	1.214
O70422	General transcription factor IIH subunit 4	Gtf2h4	52.224	1.161	1.361
O70494	Transcription factor Sp3	Sp3	82.361	1.165	1.439
O70570	Polymeric immunoglobulin receptor	Pigr	84.998	0.767	0.863
O70572	Sphingomyelin phosphodiesterase 2	Smpd2	47.466	1.344	1.159
O88286	Protein Wiz	Wiz	184.29	0.644	0.818
O88322	Nidogen-2	Nid2	153.91	1.318	1.379
O88700	Bloom syndrome protein homolog	Blm	158.36	1.681	2.059
O88746	Target of Myb protein 1	Tom1	54.325	1.278	1.999
O88792	Junctional adhesion molecule A	F11r	32.423	1.308	1.325
O88844	Isocitrate dehydrogenase [NADP] cytoplasmic	Idh1	46.674	0.764	0.856
O88947	Coagulation factor X	F10	54.017	0.884	1.632
P01027	Complement C3	C3	186.48	1.242	1.479
P02089	Hemoglobin subunit beta-2	Hbb-b2	15.878	1.465	1.396
P02463	Collagen alpha-1(IV) chain	Col4a1	160.68	1.387	1.402
P02468	Laminin subunit gamma-1	Lamc1	177.3	1.452	1.544
P02469	Laminin subunit beta-1	Lamb1	197.09	1.405	1.47
P02798	Metallothionein-2	Mt2	6.1153	0.676	0.754
P02802	Metallothionein-1	Mt1	6.0181	0.678	0.81
P03899	NADH-ubiquinone oxidoreductase chain 3	Mtnd3	13.219	1.336	1.32

P04104	"Keratin, type II cytoskeletal 1	Krt1	65.605	1.579	1.969
P05064	Fructose-bisphosphate aldolase A	Aldoa	39.355	0.789	0.745
P06151	L-lactate dehydrogenase A chain	Ldha	36.498	0.828	0.76
P06745	Glucose-6-phosphate isomerase	Gpi	62.766	0.768	0.784
P07214	SPARC OS=Mus musculus	Sparc	34.45	1.288	1.303
P08043	Zinc finger protein 2	Zfp2	52.534	0.981	0.746
P08122	Collagen alpha-2(IV) chain	Col4a2	167.32	1.398	1.355
P08228	Superoxide dismutase [Cu-Zn]	Sod1	15.942	0.685	0.794
P09602	Non-histone chromosomal protein HMG-17	Hmgn2	9.4226	0.721	1.721
P09922	Interferon-induced GTP-binding protein Mx1	Mx1	72.037	1.198	2.178
P10107	Annexin A1 OS=Mus musculus	Anxa1	38.734	1.306	1.345
P10493	Nidogen-1 OS=Mus musculus	Nid1	136.54	1.403	1.512
P11276	Fibronectin OS=Mus musculus	Fn1	272.53	1.706	1.592
P12710	"Fatty acid-binding protein, liver	Fabp1	14.245	0.749	0.838
P14152	"Malate dehydrogenase, cytoplasmic	Mdh1	36.511	0.734	0.794
P15864	Histone H1.2 OS=Mus musculus	Hist1h1c	21.266	1.401	2.705
P15920	V-type proton ATPase 116 kDa subunit a isoform 2	Atp6v0a2	98.144	1.253	1.404
P17182	Alpha-enolase	Eno1	47.14	0.796	0.732
P17665	"Cytochrome c oxidase subunit 7C, mitochondrial	Cox7c	7.3325	1.367	1.115
P17717	UDP-glucuronosyltransferase 2B17	Ugt2b17	60.855	1.328	1.279
P17809	"Solute carrier family 2, facilitated glucose transporter member 1	Slc2a1	53.984	1.379	1.05
P17897	Lysozyme C-1	Lyz1	16.794	0.684	0.795
P19137	Laminin subunit alpha-1	Lama1	338.14	1.406	1.482
P19324	Serpin H1	Serpinh1	46.533	1.523	1.6
P20152	Vimentin	Vim	53.687	1.2	1.323
P20918	Plasminogen	Plg	90.807	1.616	1.592
P22315	"Ferrochelatase, mitochondrial	Fech	47.13	1.25	1.547
P26350	Prothymosin alpha	Ptma	12.254	0.756	0.737
P27661	Histone H2AX	H2afx	15.142	1.3	2.157
P28798	Granulins	Grn	63.458	1.297	1.46
P30115	Glutathione S-transferase A3	Gsta3	25.36	0.761	0.795
P31786	Acyl-CoA-binding protein	Dbi	10	0.732	0.84
P34022	Ran-specific GTPase-activating protein	Ranbp1	23.596	0.755	0.808
P36536	GTP-binding protein SAR1a	Sar1a	22.371	1.436	2.088
P39061	Collagen alpha-1(XVIII) chain	Col18a1	182.17	1.317	1.363
P43137	Lithostathine-1	Reg1	18.518	0.554	0.678
P46412	Glutathione peroxidase 3	Gpx3	25.424	1.496	1.588
P46414	Cyclin-dependent kinase inhibitor 1B	Cdkn1b	22.193	1.379	1.261
P47915	60S ribosomal protein L29	Rpl29	17.587	1.055	1.506
P48760	"Folylpolyglutamate synthase, mitochondrial	Fpgs	64.955	1.184	1.343
P50543	Protein S100-A11	S100a11	11.083	1.286	1.318
P51670	C-C motif chemokine 9	Ccl9	13.871	0.957	2.014
P52800	Ephrin-B2	Efnb2	37.202	1.434	2.546

P54227	Stathmin	Stmn1	17.274	0.665	0.747
P54754	Ephrin type-B receptor 3	Ephb3	109.66	1.164	1.722
P55050	Fatty acid-binding protein	Fabp2	15.126	0.747	0.817
P55821	Stathmin-2 OS=Mus musculus	Stmn2	20.828	0.607	0.671
P55937	Golgin subfamily A member 3	Golga3	167.22	1.125	1.337
P58044	Isopentenyl-diphosphate Delta-isomerase 1	Idi1	26.289	0.756	0.74
P59326	YTH domain-containing family protein 1	Ythdf1	60.878	1.177	1.411
P60904	DnaJ homolog subfamily C member 5	Dnajc5	22.101	1.257	1.377
P61022	Calcineurin B homologous protein 1	Chp1	22.432	1.327	1.159
P61961	Ubiquitin-fold modifier 1	Ufm1	9.1175	0.77	0.734
P61965	WD repeat-containing protein 5	Wdr5	36.588	1.149	1.422
P61971	Nuclear transport factor 2	Nutf2	14.478	0.811	0.767
P62313	U6 snRNA-associated Sm-like protein LSm6	Lsm6	9.1275	0.828	0.701
P62342	Thioredoxin reductase-like selenoprotein T	Selenot	22.292	1.39	1.128
P62627	Dynein light chain roadblock-type 1	Dynlrb1	10.99	0.761	0.777
P62858	40S ribosomal protein S28	Rps28	7.8409	1.178	1.654
P62984	Ubiquitin-60S ribosomal protein L40	Uba52	14.728	1.075	1.347
P63166	Small ubiquitin-related modifier 1	Sumo1	11.557	1.073	1.855
P63254	Cysteine-rich protein 1	Crip1	8.5497	0.716	0.689
P68037	Ubiquitin-conjugating enzyme E2 L3	Ube2l3	17.861	0.767	0.822
P84228	Histone H3.2	Hist1h3b	15.388	1.096	1.721
P84244	Histone H3.3	H3f3a	15.328	1.305	1.869
P97466	Noggin	Nog	25.77	1.285	2.294
P97789	5'-3' exoribonuclease 1	Xrn1	194.31	1.284	1.763
P97872	Dimethylaniline monooxygenase [N-oxide-forming] 5	Fmo5	60	1.281	1.321
Q01237	3-hydroxy-3-methylglutaryl-coenzyme A reductase	Hmgcr	97.039	1.401	1.105
Q05793	Basement membrane-specific heparan sulfate proteoglycan core protein	Hspg2	398.29	1.332	1.434
Q08879	Fibulin-1	Fbln1	78.032	1.29	1.521
Q31125	Zinc transporter SLC39A7	Slc39a7	50.656	1.236	1.396
Q3TKY6	Spliceosome-associated protein CWC27 homolog	Cwc27	53.542	0.757	0.775
Q3TMQ6	Angiogenin-4	Ang4	16.425	0.63	0.717
Q3U1G5	Interferon-stimulated 20 kDa exonuclease-like 2	Isg20l2	41.019	1.047	1.325
Q3UMU9	Hepatoma-derived growth factor-related protein 2	Hdgfl2	74.29	0.977	1.325
Q3UUQ7	GPI inositol-deacylase	Pgap1	104.58	1.305	1.186
Q3UX10	Tubulin alpha chain-like 3	Tubal3	49.988	1.114	1.345
Q45VN2	Alpha-defensin 20	Defa20	10.601	0.713	0.854
Q4VAE3	Transmembrane protein 65	Tmem65	24.918	1.415	1.358
Q4ZJM7	Otolin-1 OS=Mus musculus	Otol1	49.6	1.284	2.128
Q5G865	Alpha-defensin 24	Defa24	10.327	1.449	2.702
Q5I012	Putative sodium-coupled neutral amino acid transporter 10	Slc38a10	117.19	1.113	1.374
Q5SVR0	TBC1 domain family member 9B	Tbc1d9b	141.78	0.729	0.945

Q60592	Microtubule-associated serine/threonine-protein kinase 2	Mast2	190.53	1.51	1.458
Q60722	Transcription factor 4	Tcf4	71.624	1.32	1.413
Q60829	Protein phosphatase 1 regulatory subunit 1B	Ppp1r1b	21.78	0.714	0.828
Q61152	Tyrosine-protein phosphatase non-receptor type 18	Ptpn18	50.201	1.034	1.315
Q61292	Laminin subunit beta-2	Lamb2	196.58	1.335	1.409
Q61382	TNF receptor-associated factor 4	Traf4	53.503	1.067	1.445
Q61598	Rab GDP dissociation inhibitor beta	Gdi2	50.537	0.754	0.804
Q61749	Translation initiation factor eIF-2B subunit delta	Eif2b4	57.624	1.149	1.378
Q62203	Splicing factor 3A subunit 2	Sf3a2	49.911	1.078	1.722
Q62241	U1 small nuclear ribonucleoprotein C	Snrpc	17.364	0.723	0.931
Q62266	Cornifin-A OS=Mus musculus	Sprr1a	15.765	1.498	1.214
Q62273	Sulfate transporter	Slc26a2	81.603	1.29	1.489
Q62313	Trans-Golgi network integral membrane protein 1	Tgoln1	37.848	1.32	1.398
Q62388	Serine-protein kinase ATM	Atm	349.41	1.068	1.427
Q62392	Pleckstrin homology-like domain family A member 1	Phlda1	45.582	1.403	1.203
Q62395	Trefoil factor 3 OS=Mus musculus	Tff3	8.8081	0.728	0.866
Q62452	UDP-glucuronosyltransferase 1-9	Ugt1a9	60.007	1.338	1.626
Q64435	UDP-glucuronosyltransferase 1-6	Ugt1a6	60.438	1.213	1.308
Q64458	Cytochrome P450 2C29	Cyp2c29	55.715	1.286	1.376
Q64459	Cytochrome P450 3A11	Cyp3a11	57.854	1.528	1.662
Q64464	Cytochrome P450 3A13	Cyp3a13	57.492	1.428	1.29
Q66JX5	FGFR1 oncogene partner	Fgfr1op	42.758	0.722	0.889
Q689Z5	Protein strawberry notch homolog 1	Sbno1	153.74	1.044	1.412
Q6NVG1	Lysophospholipid acyltransferase LPCAT4	Lpcat4	57.143	1.44	1.219
Q6PDH0	Pleckstrin homology-like domain family B member 1	Phldb1	150.07	0.621	0.558
Q6PGC1	ATP-dependent RNA helicase DHX29	Dhx29	153.97	1.247	1.635
Q6PHN9	Ras-related protein Rab-35 OS=Mus musculus	Rab35	23.025	1.236	1.347
Q6PIJ4	Nuclear factor related to kappa-B-binding protein	Nfrkb	138.76	1.125	1.673
Q6ZQ06	Centrosomal protein of 162 kDa	Cep162	160.85	1.088	2.187
Q6ZQF0	DNA topoisomerase 2-binding protein 1	Topbp1	168.86	1.271	1.31
Q6ZWY9	Histone H2B type 1-C/E/G	Hist1h2bc	13.906	1.383	4.136
Q71KT5	Delta(14)-sterol reductase	Tm7sf2	46.52	1.489	1.611
Q71LX4	Talin-2 OS=Mus musculus	Tln2	253.62	1.024	1.663
Q76KJ5	DNA-directed RNA polymerase I subunit RPA34 OS=Mus musculus	Cd3eap	43.082	0.977	1.393
Q78IK2	Up-regulated during skeletal muscle growth protein 5	Usmg5	6.3814	1.429	1.374
Q791T5	Mitochondrial carrier homolog 1	Mtch1	41.565	1.198	2.201
Q7TNS2	MICOS complex subunit Mic10	Minos1	8.5669	1.496	1.349
Q7TT45	Ras-related GTP-binding protein D	Rragd	51.232	1.206	1.545
Q80T69	Lysine-specific demethylase 9	Rsbn1	89.25	1.466	1.23

Q80TE0	RNA polymerase II-associated protein 1	Rpap1	155.27	1.275	1.806
Q80U49	Centrosomal protein of 170 kDa protein B	Cep170b	170.82	1.087	1.358
Q80WQ6	Inactive rhomboid protein 2	Rhbdf2	93.433	1.102	1.414
Q80X80	Phospholipid transfer protein C2CD2L	C2cd2l	76.328	1.376	1.385
Q80YT7	Myomegalin	Pde4dip	250.63	1.584	1.494
Q80ZM8	Cardiolipin synthase (CMP-forming)	Crls1	32.502	1.219	1.308
Q80ZW2	Protein THEM6	Them6	23.802	1.4	1.385
Q8BG73	SH3 domain-binding glutamic acid-rich-like protein 2	Sh3bgrl2	12.255	0.86	0.753
Q8BH59	Calcium-binding mitochondrial carrier protein Aralar1	Slc25a12	74.569	1.297	1.343
Q8BHE8	"m-AAA protease-interacting protein 1, mitochondrial	Maip1	32.985	1.412	1.37
Q8BHG2	UPF0587 protein C1orf123 homolog	---	18.02	0.768	0.85
Q8BHL4	Retinoic acid-induced protein 3	Gprc5a	40.1	1.51	1.204
Q8BIG7	Catechol O-methyltransferase domain-containing protein 1	Comtd1	28.96	1.346	1.17
Q8BJ03	Cytochrome c oxidase assembly protein COX15 homolog	Cox15	45.852	1.317	1.144
Q8BMD8	Calcium-binding mitochondrial carrier protein SCaMC-1	Slc25a24	52.901	1.367	1.24
Q8BND5	Sulfhydryl oxidase 1	Qsox1	82.784	0.757	0.844
Q8BNU0	Armadillo repeat-containing protein 6	Armc6	50.683	1.303	1.705
Q8BNW9	Kelch repeat and BTB domain-containing protein 11	Kbtbd11	67.945	1.227	1.331
Q8BP92	Reticulocalbin-2	Rcn2	37.27	1.26	1.407
Q8BQZ4	Ral GTPase-activating protein subunit beta	Ralgapb	165.2	1.232	1.738
Q8BTU1	Cilia- and flagella-associated protein 20	Cfap20	22.748	1.06	1.327
Q8BTW3	Exosome complex component MTR3	Exosc6	28.37	1.244	1.937
Q8BW75	Amine oxidase [flavin-containing] B OS=Mus musculus	Maob	58.557	1.377	1.287
Q8BWQ1	UDP-glucuronosyltransferase 2A3	Ugt2a3	61.119	1.337	1.306
Q8BZR9	Nuclear cap-binding protein subunit 3	Ncbp3	70.042	1.312	1.568
Q8C3B8	Protein RFT1 homolog	Rft1	60.303	1.24	1.368
Q8C4V1	Rho GTPase-activating protein 24	Arhgap24	84.099	0.809	1.663
Q8C561	LMBR1 domain-containing protein 2	Lmbrd2	81.1	1.293	1.335
Q8C5T8	Coiled-coil domain-containing protein 113	Ccdc113	44.214	0.876	0.761
Q8CD91	SPARC-related modular calcium-binding protein 2	Smoc2	49.891	0.873	1.421
Q8CGA0	Protein phosphatase 1F	Ppm1f	49.61	0.665	0.975
Q8CHP8	Glycerol-3-phosphate phosphatase	Pgp	34.54	0.757	0.835
Q8CIM7	Cytochrome P450 2D26	Cyp2d26	56.975	1.215	1.359
Q8JZL7	Ras-GEF domain-containing family member 1B	Rasgef1b	55.273	1.201	1.425
Q8JZR0	Long-chain-fatty-acid--CoA ligase 5	Acsl5	76.205	1.316	1.306
Q8K072	Receptor expression-enhancing protein 4	Reep4	29.69	1.194	1.43
Q8K0C5	Zymogen granule membrane protein 16	Zg16	18.209	1.48	1.314

Q8K0E3	Sodium/myo-inositol cotransporter 2	Slc5a11	73.797	1.165	1.305
Q8K0E8	Fibrinogen beta chain	Fgb	54.752	1.419	1.493
Q8K296	Myotubularin-related protein 3	Mtmr3	133.84	0.909	1.402
Q8K2C9	Very-long-chain (3R)-3-hydroxyacyl-CoA dehydratase 3	Hacd3	43.131	1.31	1.379
Q8K3Z0	Nucleotide-binding oligomerization domain-containing protein 2	Nod2	113.56	0.692	0.679
Q8R003	Muscleblind-like protein 3	Mbnl3	37.57	1.156	1.497
Q8R1M8	Mucosal pentraxin	Mptx1	24.538	1.307	1.236
Q8R2K1	Fucose mutarotase	Fuom	16.805	0.766	0.72
Q8R3P6	Integrator complex subunit 14	Ints14	57.236	1.292	1.384
Q8R4D5	Transient receptor potential cation channel subfamily M member 8	Trpm8	127.71	0.992	2.019
Q8VCM7	Fibrinogen gamma chain	Fgg	49.391	1.366	1.378
Q8VED9	Galectin-related protein	Lgalsl	18.955	1.274	1.718
Q8VHG0	Dimethylaniline monooxygenase [N-oxide-forming] 4	Fmo4	63.791	1.328	1.268
Q8VHK1	Caskin-2	Caskin2	126.78	1.109	1.786
Q91V04	Translocating chain-associated membrane protein 1	Tram1	43.039	1.249	1.349
Q91V76	Ester hydrolase C11orf54 homolog	---	34.995	0.8	0.763
Q91W97	Putative hexokinase HKDC1	Hkdc1	102.26	1.365	1.25
Q91WE4	UPF0729 protein C18orf32 homolog	---	8.0355	1.485	2.253
Q91WP6	Serine protease inhibitor A3N	Serpina3n	46.717	1.605	1.586
Q91XB7	Protein YIF1A	Yif1a	32.134	1.384	1.404
Q91Y74	"CMP-N-acetylneuraminate-beta-galactosamide-alpha-2,3-sialyltransferase 4	St3gal4	38.058	1.429	1.198
Q91ZF2	Cathepsin 7	Cts7	37.724	1.398	1.782
Q921I1	Serotransferrin	Tf	76.723	1.277	1.36
Q921U8	Smoothelin	Smtn	100.29	1.376	1.889
Q923S9	Ras-related protein Rab-30	Rab30	23.058	1.615	1.409
Q93092	Transaldolase	Taldo1	37.387	0.755	0.742
Q99JR5	Tubulointerstitial nephritis antigen-like	Tinagl1	52.664	1.455	1.4
Q99LH1	Nucleolar GTP-binding protein 2	Gnl2	83.345	1.006	1.419
Q99LJ0	CTTNBP2 N-terminal-like protein	Cttnbp2nl	69.84	1.085	1.339
Q99LX0	Protein/nucleic acid deglycase DJ-1	Park7	20.021	0.722	0.794
Q99MI1	ELKS/Rab6-interacting/CAST family member 1	Erc1	128.33	1.014	1.497
Q99PG0	Arylacetamide deacetylase	Aadac	45.25	1.303	1.19
Q9CQC2	Colipase OS=Mus musculus	Clps	12.444	0.608	0.747
Q9CQD1	Ras-related protein Rab-5A	Rab5a	23.598	1.329	1.5
Q9CQJ8	NADH dehydrogenase [ubiquinone] 1 beta subcomplex subunit 9 OS=Mus musculus OX=10090 GN=Ndufb9 PE=1 SV=3	Ndufb9	21.984	1.317	1.27
Q9CQM2	ER lumen protein-retaining receptor 2	Kdelr2	24.454	1.353	3.241
Q9CQS5	Serine/threonine-protein kinase RIO2	Riok2	62.49	1.175	2.177

Q9CQT2	RNA-binding protein 7	Rbm7	30.148	1.059	1.405
Q9CR62	Mitochondrial 2-oxoglutarate/malate carrier protein	Slc25a11	34.155	1.352	1.277
Q9CWK3	CD2 antigen cytoplasmic tail-binding protein 2	Cd2bp2	37.694	0.995	1.419
Q9CWV6	PRKR-interacting protein 1	Prkrip1	21.491	1.159	1.654
Q9CWY9	RPA-interacting protein	Rpain	24.897	1.557	2.202
Q9CXY6	Interleukin enhancer-binding factor 2	Ilf2	43.062	0.755	0.789
Q9CY57	Chromatin target of PRMT1 protein	Chtop	26.585	0.753	0.842
Q9CYH2	Redox-regulatory protein FAM213A	Fam213a	24.394	1.404	1.414
Q9D032	Single-stranded DNA-binding protein 3	Ssbp3	40.421	1.225	1.333
Q9D136	2-oxoglutarate and iron-dependent oxygenase domain-containing protein 3	Ogfod3	35.384	1.273	1.559
Q9D1I2	Caspase recruitment domain-containing protein 19	Card19	20.936	1.348	1.349
Q9D1J1	Adaptin ear-binding coat-associated protein 2	Necap2	28.598	0.968	1.591
Q9D1N9	"39S ribosomal protein L21, mitochondrial	Mrpl21	23.366	1.019	1.38
Q9D2L9	Protein FAM111A	Fam111a	69.948	1.137	1.309
Q9D2Q2	Probable tRNA (uracil-O(2)-)-methyltransferase	Trmt44	79.835	1.257	1.399
Q9D379	Epoxide hydrolase 1	Ephx1	52.576	1.318	1.336
Q9D6M3	Mitochondrial glutamate carrier 1	Slc25a22	34.67	1.112	1.487
Q9D7S0	Ly6/PLAUR domain-containing protein 8	Lypd8	27.524	0.713	0.684
Q9DB90	Protein SMG9	Smg9	57.62	1.035	1.372
Q9DBC3	Cap-specific mRNA (nucleoside-2'-O-)-methyltransferase 1	Cmtr1	95.675	0.667	0.933
Q9DBM1	G patch domain-containing protein 1	Gpatch1	103.01	1.045	1.308
Q9DBT3	Coiled-coil domain-containing protein 97	Ccdc97	38.724	1.235	1.477
Q9DBY1	E3 ubiquitin-protein ligase synoviolin	Syvn1	67.296	1.419	2.27
Q9DCF9	Translocon-associated protein subunit gamma	Ssr3	21.064	1.32	2.09
Q9DCS9	NADH dehydrogenase [ubiquinone] 1 beta subcomplex subunit 10	Ndufb10	21.024	1.43	1.319
Q9DCT5	Stromal cell-derived factor 2	Sdf2	23.159	1.715	2.419
Q9EP75	Leukotriene-B4 omega-hydroxylase 3	Cyp4f14	59.8	1.388	1.328
Q9EPB4	Apoptosis-associated speck-like protein containing a CARD	Pycard	21.458	0.72	0.728
Q9EPS3	D-glucuronyl C5-epimerase	Glce	70.088	1.303	1.298
Q9EQ06	Estradiol 17-beta-dehydrogenase 11	Hsd17b11	32.88	1.363	1.371
Q9JKP5	Muscleblind-like protein 1	Mbnl1	36.975	1.203	1.534
Q9JLJ1	Selenoprotein K	Selenok	10.642	1.426	1.302
Q9JLJ5	Elongation of very long chain fatty acids protein 1	Elovl1	32.677	1.637	2.101
Q9JM52	Misshapen-like kinase 1	Mink1	147.29	1.213	1.663
Q9QXD8	LIM domain-containing protein 1	Limd1	71.421	1.248	1.831
Q9QYI4	DnaJ homolog subfamily B member 12	Dnajb12	41.987	1.331	1.616
Q9QZI9	Serine incorporator 3	Serinc3	52.622	1.127	1.55
Q9QZR0	E3 ubiquitin-protein ligase RNF25	Rnf25	51.226	0.737	0.794
Q9R0B9	"Procollagen-lysine,2-oxoglutarate 5-dioxygenase 2	Plod2	84.487	1.421	1.469
Q9R0E1	"Procollagen-lysine,2-oxoglutarate 5-dioxygenase 3	Plod3	84.921	1.298	1.306

Q9R0Q3	Transmembrane emp24 domain-containing protein 2	Tmed2	22.705	1.259	1.333
Q9R0U0	Serine/arginine-rich splicing factor 10	Srsf10	31.3	1.127	1.345
Q9WTI7	Unconventional myosin-Ic	Myo1c	121.94	1.335	1.267
Q9WUZ9	Ectonucleoside triphosphate diphosphohydrolase 5	Entpd5	47.101	1.247	1.318
Q9WVQ5	Methylthioribulose-1-phosphate dehydratase	Apip	26.949	0.754	0.914
Q9Z1S5	Neuronal-specific septin-3	Sept3	40.037	0.75	1.118
Q9Z247	Peptidyl-prolyl cis-trans isomerase FKBP9	Fkbp9	62.995	1.388	1.373
Q9Z2A7	Diacylglycerol O-acyltransferase 1	Dgat1	56.789	1.253	1.406
Q9Z2G0	Protein fem-1 homolog B	Fem1b	70.222	1.49	1.777
Q9Z2Z6	Mitochondrial carnitine/acylcarnitine carrier protein	Slc25a20	33.026	1.318	1.194
